# Neuroprotective Properties of* Panax notoginseng* Saponins via Preventing Oxidative Stress Injury in SAMP8 Mice

**DOI:** 10.1155/2017/8713561

**Published:** 2017-01-30

**Authors:** Jin-Lan Huang, Xin Jing, Xin Tian, Mei-Chun Qin, Zhe-Hao Xu, Deng-Pan Wu, Zhen-Guo Zhong

**Affiliations:** ^1^Jiangsu Key Laboratory of New Drug Research and Clinical Pharmacy, Pharmacy School, Xuzhou Medical University, Xuzhou, Jiangsu 221004, China; ^2^Department of Pharmacology, Pharmacy School, Xuzhou Medical University, Xuzhou, Jiangsu 221004, China; ^3^Department of Pharmacology, Xi'an Medical College, Xi'an, Shangxi 710309, China; ^4^Guangxi Adverse Drug Reaction Monitoring Center, Nanning, Guangxi 530029, China; ^5^Department of Science and Technology, Guangxi University of Chinese Medicine, Nanning, Guangxi 530200, China

## Abstract

Inhibiting oxidative damage in early stage of Alzheimer's disease (AD) is considered as a strategy for AD treatment. Our previous study has shown that* Panax notoginseng* saponins (PNS) have an antiaging action by increasing the levels of superoxide dismutase (SOD), catalase (CAT), and glutathione peroxidase (GSH-PX) in the serum of aged rats. In this study, we aimed to investigate the effects of PNS on antioxidant enzymes and uncoupling proteins (UCPs) involved in oxidative stress in AD mice. The results showed that PNS prevented neuronal loss in hippocampal CA1 region and alleviated pathomorphological change of neurons in CA1 region. Moreover, PNS inhibited the production of 8-hydroxydeoxyguanosine (8-OHdG), enhanced the expressions and activities of SOD, CAT, and GSH-PX, and improved the mRNA and protein levels of UCP4 and UCP5 in the brains of SAMP8 mice. Together, our study shows that PNS has the ability to protect neurons in AD brain from oxidative stress damage through attenuating the production of 8-OHdG, enhancing the activities of antioxidant enzymes and the expressions levels of UCP4 and UCP5. Accordingly, PNS may be a promising agent for AD treatment.

## 1. Introduction

Alzheimer's disease (AD) is an age-related neurodegenerative disorder and defined clinically by a progressive decline in cognitive function and neuropathologically by the presence of neurofibrillary tangles and neuritic plaques. It has been suggested that oxidative imbalance and resultant neuronal damage may play a critical role in the initiation and progression of AD [1]. Hence, inhibiting oxidative damage in the early stage of AD is considered as a strategy for AD treatment.

Oxidative damage in the CNS of AD patients may be attributed to the increased production of free radicals or the failure of antioxidant defenses. Free radicals have been reported to attack deoxyribonucleic acid, leading to the overproduction of 8-hydroxydeoxyguanosine (8-OHdG). In some areas of the brain during the early stage of AD pathology, significant increment in 8-OHdG has been observed [2]. Additionally, the impairment of antioxidant defense, in which antioxidant enzymes such as superoxide dismutase (SOD), catalase (CAT), and glutathione peroxidase (GSH-PX) are included, was found in postmortem brain tissue of AD patients. Body of data suggests that the activities of SOD, CAT, and GSH-PX reduce in AD hippocampus [3]. Furthermore, the uncoupling protein 4 (UCP4) and UCP5, which are considered to be able to prevent excessive mitochondrial reactive oxygen species (ROS) production [4], have been reported to play a profound role in preventing oxidative stress damage [5]. Therefore, antioxidants targeting antioxidant enzymes and uncoupling proteins (UCPs) may have potentials to alleviate oxidative damage, thus preventing the exacerbations of AD at the early stage of the disease.

*Panax notoginseng*, known as Sanqi in Chinese, is mainly distributed in the Yunnan province and Guangxi Zhuang Autonomous Region in China. The root of* Panax notoginseng* has plenty of medicinal properties including blood clotting, alleviating pain and relieving swelling [6].* Panax notoginseng* saponins (PNS) are the main active compound extracted from the root of* Panax notoginseng*. A study shows that PNS contains about 20 saponin constituents, and ginsenosides Rb1 and Rg1 comprise a large proportion of the constituents [7]. It has been reported that ginsenosides Rb1 and Rg1 have neuroprotective actions [8]. Our previous studies showed that PNS could remarkably improve learning and memory performance and retard the deposition of amyloid *β*-peptide (A*β*) 1–40 and A*β*1–42 by the expression of *β*-amyloid precursor protein (APP) in senescence-accelerated mouse-prone 8 (SAMP8) [9–11]. Recently, our study has shown that PNS has an antiaging action by increasing the levels of SOD, CAT, and GSH-PX in the serum of aged rats [12]. Accordingly, it is reasonable to speculate that PNS may have an antagonistic effect on oxidative stress injury in the early-stage AD.

SAMP8 mouse is regarded as a model of AD-like dementia with high oxidative stress-related impairments [13] and shows age-dependent memory deficits, neuropathology, and increased amyloid plaque load, thereby being considered as a model of sporadic AD [14]. There are reports showing that lipid peroxidation caused by oxidative stress is increased in SAMP8 mice as early as 2 months of age [15], and apparent memory deficits, increased amyloid plaque production, and remarkable reductions in the activities of antioxidant enzymes including CAT and GSH-PX were found in 5-month-old SAMP8 mice [16]. Therefore, in the present study, 3-month-old SAMP8 mice were intragastrically given PNS for 8 consecutive weeks to characterize the impact of PNS on antioxidant enzymes and UCPs involved in oxidative stress, thereby providing a new opportunity for research with respect to the pharmaceutical prevention and treatment of AD.

## 2. Materials and Methods

### 2.1. Drugs and Reagents

PNS was obtained from Yunnan Yunke Pharmaceutical Manufacture Co., Ltd. (Yunnan, China). Huperzine A (Hup A) was from Forward Group (Shanghai, China). The SOD, CAT, and GSH-PX activity kits were purchased from Nanjing Jiancheng Bioengineering Institute (Nanjing, China). Mouse 8-OHdG ELISA kit was from Vicmed Biotech Co., Ltd. (Xuzhou, China). SOD, CAT, GSH-PX, UCP4, and UCP5 antibodies were from Santa Cruz Biotechnology, Inc. (Texas, USA). *β*-Actin antibody was from Arigo biolaboratories (Hsinchu, China). Horseradish peroxide- (HRP-) labeled goat-anti-rabbit and goat-anti-mouse (for GSH-PX) IgG working solution and DAB staining solution were obtained from Beijing Zhongshan Golden Bridge Biotechnology Co., Ltd. (Beijing, China). IRDye 800CW goat anti-mouse IgG and goat anti-rabbit IgG were from LI-COR Biotechnology, Inc. (Nebraska, USA). The ReverTra Ace® qPCR RT Kit was obtained from Toyobo Life Science Department (Osaka, Japan).

### 2.2. Animals, Drug Treatment, and Tissue Preparation

Sixty 3-month-old SAMP8 and fifteen 3-month-old senescence-accelerated-resistant (SAMR1) pathogen- and virus-free mice, which are considered to be the control-strain of SAMP8, were purchased from Beijing HFK Bioscience Co., Ltd. (Beijing, China). These mice were randomly divided into four groups: model group, PNS high-dosage, PNS low-dosage groups, and Hup A group (the positive control). SAMR1 mice were considered as the control group. Drug treatment was performed according to our previous study [11]. Briefly, the low- and high-dosage groups were intragastrically given 100 and 200 mg/kg of PNS everyday, respectively, while Hup A was given by gavage to the mice in the Hup A group at 0.3 mg/kg for 8 consecutive weeks. The same volume of distilled water was provided to the model and control groups. The mice were euthanatized while the brains were excised for immunohistochemistry staining, hematoxylin and eosin staining, activity detection of antioxidant enzymes, western blotting, and real-time PCR assay. Animal care and experimental procedures were implemented in accordance with the document “Guidance Suggestions for Caring for Laboratory Animals” produced by the Ministry of Science and Technology of China in 2006.

### 2.3. Hematoxylin and Eosin (HE) Staining

Paraffin-embedded brain tissues were sliced and the sections were deparaffinized at routine, washed by distilled water, stained in hematoxylin solution, washed by acid alcohol and running water, counterstained in eosin solution, dehydrated through graded alcohol, and mounted with neutral gum. The neuronal pathological changes were observed using light microscope (Olympus BX-50, Japan). Imaging-Pro-Plus 6.0 software (Media Cybernetics Inc., America) was used to perform quantitative analysis of neuronal numbers. The neurons in CA1 region of each section were counted in 10 different high powered fields (HPF, 400x) and 3 to 5 serial sections of each sample were used to do the count.

### 2.4. 8-OHdG Detection

The level of 8-OHdG was detected according to 8-OHdG ELISA kit instruction. Briefly, sample diluents or standard solution was mixed with a horseradish peroxidase-conjugate reagent. The optical density was read at 450 nm using visible spectrophotometer. The standard curve was generated and the concentration of 8-OHdG in the samples was determined by comparing the OD values of the samples to the standard curve.

### 2.5. SOD, CAT, and GSH-PX Activity Detection

SOD, CAT, and GSH-PX activities were detected according to SOD, CAT, and GSH activity assay kit instructions, respectively. Briefly, action buffers were added to the supernatants of tissue samples. The mixtures were incubated at 37°C and the optical densities were measured at 550 nm, 412 nm, and 405 nm using visible spectrophotometer for SOD, GSH-PX, and CAT activities, respectively. The activities can be expressed as the units per mg of protein sample.

### 2.6. Immunohistochemistry Staining

Paraffin-embedded brain tissue was prepared and the sections were incubated with primary antibodies (dilution 1 : 200 for SOD, CAT and GSH-PX) at 4°C overnight, followed by incubation with horseradish peroxide- (HRP-) labeled goat-anti-rabbit (for SOD and CAT) and goat-anti-mouse (for GSH-PX) IgG working solution. The sections were then stained in DAB staining solution, restained with hematoxylin, gradient dehydrated, and photographed using microscope (Olympus BX-50, Japan). The percentage of immunopositive neuron in hippocampus was calculated using the following equation: immunopositive neuron (%) = the number of immunopositive neurons/the number of total neurons in each fields × 100.

### 2.7. Western Blotting Assay

The extracted protein samples were separated by SDS-polyacrylamide gel electrophoresis in 10% Tris-glycine gels and transferred to a nitrocellulose transfer membrane (Excell Bio, Shanghai, China). Primary antibodies including UCP4 (dilution 1 : 500), UCP5 (dilution 1 : 500), and *β*-actin (dilution 1 : 4000) were incubated at 4°C overnight. Secondary antibodies were IRDye 800CW purified immunoglobulin-conjugated anti-rabbit (dilution 1 : 10000). Immunopositive bands were visualized at Ex/Em = 778 nm/795 nm.

### 2.8. Extraction of mRNA, Reverse Transcription, and Real-Time PCR

The reverse transcription reaction system contained 2 *μ*g total RNA, 1 *μ*L RT Enzyme Mix, 4 *μ*L 5x RT Buffer, 1 *μ*L Primer Mix, and RNase free water. The real-time PCR reaction system was a 10 *μ*L reaction mixture containing 0.5 *μ*L cDNA, 4.1 *μ*L RNase-free water, 5 *μ*L 2x SYBR Green Master Mix, and 1.8 *μ*L of each primer (10 mM). The reactions were performed under the following cycling conditions: initial denaturation at 95°C for 10 min, followed by 45 cycles of 95°C for 15 s, and 60°C for 30 s with a final extension at 72°C for 5 min.

The primer sequences were as follows: ACTB (174 bp), sense (5′-GTGCTATGTTGCTCTAGACTTCG-3′), UCP4 (132 bp), sense (5′-TCGAGACAAACAAGGAAGGGG-3′), antisense (5′-GACCAAGGGGTCATTCTCAGC-3′); and UCP5 (103 bp), sense (5′-CCGTCGGTTTCAATGCTTCC-3′), and antisense (5′-GAAGGGACTCAGCTGCTCAA-3′). The relative gene expression was calculated by 2^−ΔΔCp^ method, where ΔΔCp = ΔCp^treatment^ − ΔCp^control^ and ΔCp = Cp^target  gene^ − Cp^*ATCB*  gene^ [17].

### 2.9. Statistical Analysis

The data in each group were presented as mean ± SD. Analysis of variance was implemented using the SPSS software (IBM, New York, USA) for Windows 13.0 using one-way analysis of variance. *P* < 0.05 was considered statistically significant.

## 3. Results

### 3.1. Effects of PNS on Pathomorphological Changes in the Hippocampus of SAMP8 Mice

**Figure 1:**
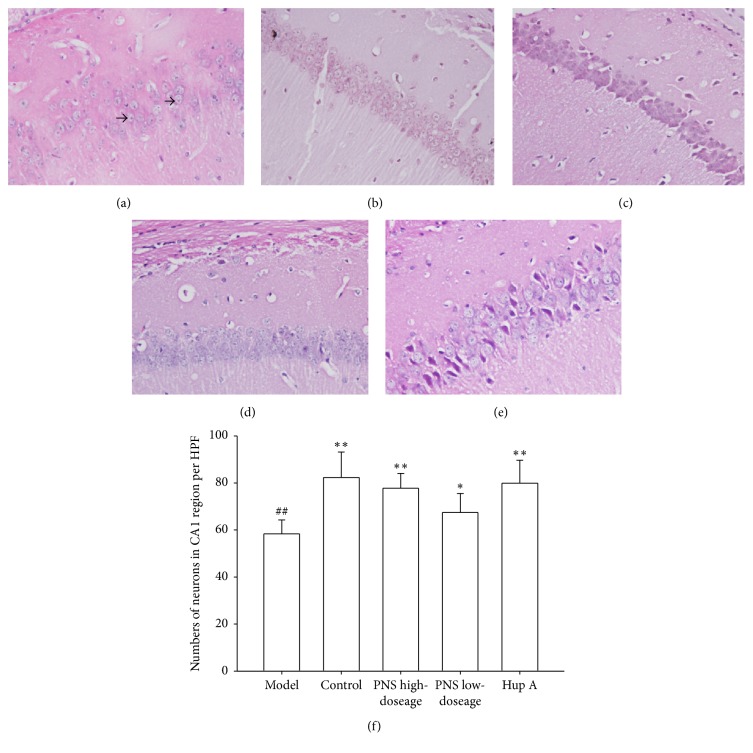
Hematoxylin and eosin (HE) staining of representative sections of hippocampal CA1 region under a 400x light microscopy (scale bar = 25 *μ*m); the karyopyknosis in the model group was marked with arrows. (a–e) HE staining of representative sections of the model, control, PNS high-dosage, PNS low-dosage, and Hup A (positive control) group, respectively; (f) neuronal numbers of each section were quantitatively analyzed in 10 high powered fields (HPF, 400x) using Imaging-Pro-Plus software 6.0. Data represent mean ± SD from 4-5 independent samples. Statistically significant differences were calculated by one-way ANOVA using the SPSS 13.0 software. ^##^*P* < 0.01, versus control group; ^*∗*^*P* < 0.05, ^*∗∗*^*P* < 0.01, versus model group.

It has been established that the hippocampus is one of the main damaged regions of the brain in AD and the neurons in hippocampal CA1 region are susceptible to oxidative stress and vulnerable to the injury induced by oxidative stress [18]. Thus, in the study, the hippocampal neurons in CA1 region were chosen to be stained by HE and the morphology of neurons in CA1 region was observed using 400x light microscopy. As illustrated in Figures 1(a)–1(e), in the control group, the hippocampal neurons in CA1 region were arranged in an organized pattern with clear boundaries, whereas the CA1 region neurons of the model group exhibited irregular and loose arrangements and showed condensed nucleus. The CA1 region in PNS treatment groups displayed different appearance, in which the neurons were arranged regularly and densely and fewer karyopyknotic neurons were observed (Figures 1(a)–1(e)). Moreover, the number of HE-stained neurons in CA1 region was assessed using Imaging-Pro-Plus 6.0 software. The results showed that the neuronal numbers in CA1 region in the control and PNS treatment groups were significantly higher than model group (Figure 1(f)), indicating that PNS treatment has ability to prevent neuronal loss.

### 3.2. Effect of PNS on 8-OHdG Production

**Figure 2:**
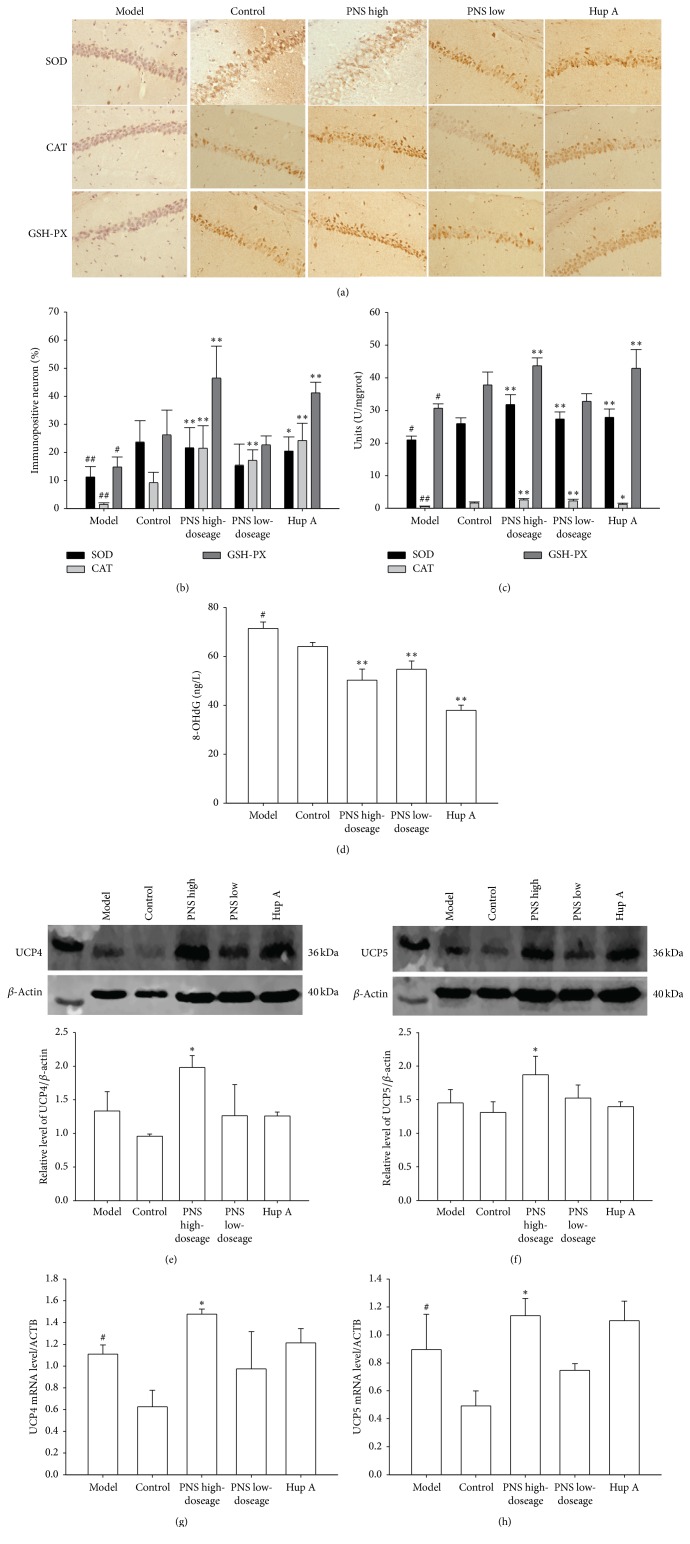
Effects of PNS on oxidative stress injury in SAMP8 mice. (a) Immunohistochemical analysis of superoxide dismutase (SOD), catalase (CAT), and glutathione peroxidase (GSH-PX) expressions in the hippocampal CA1 region, respectively (scale bar = 25 *μ*m); (b and c) the percentages of immunopositive neurons and activities of SOD, CAT, and GSH-PX, respectively; (d) 8-hydroxydeoxyguanosine (8-OHdG) production; (e and f) uncoupling protein 4 (UCP4) and UCP5 protein expressions, respectively; (g and h) UCP4 and UCP5 mRNA expressions, respectively. Data in each experiment represent mean ± SD from 4-5 independent samples. Statistically significant differences were calculated by one-way ANOVA using the SPSS 13.0 software. ^#^*P* < 0.05 and ^##^*P* < 0.01, versus control group; ^*∗*^*P* < 0.05 and ^*∗∗*^*P* < 0.01, versus model group.

As shown in Figure 2(d), the 8-OHdG levels in the control and PNS treatment groups were significantly lower than in the model group, suggesting that PNS hold a potential to inhibit the production of 8-OHdG in SAMP8 mice.

### 3.3. Effects of PNS on SOD, CAT, and GSH Expressions and Activities

As illustrated in Figures 2(a)–2(c), the percentages of immunopositive neurons in hippocampus and activities of SOD, CAT, and GSH-PX were remarkably lower in the model group than in the control group. After SAMP8 mice were treated with high dose of PNS, the percentages of immunopositive neurons and activities of SOD, CAT, and GSH-PX were significantly enhanced.

### 3.4. Effects of PNS on the Levels of UCP4 and UCP5 mRNA and Protein Expression

As shown in Figures 2(e)–2(h), the levels of UCP4 and 5 mRNA, rather than UCP4 and UCP5 protein expression in the model group, were remarkably higher than that in the control group. In addition, high-dosage PNS treatment induced a significant increase in both mRNA and protein expressions.

## 4. Discussion

It has been suggested that neurons in hippocampal CA1 region are highly vulnerable to oxidative stress [19], which may result in neuronal loss and morphological change. The histopathological findings indicated that the neurons in hippocampal CA1 region in the model group were arranged irregularly and loosely and showed pycnotic nucleus. PNS treatment significantly reversed these pathomorphological changes. For example, neuronal cells exhibited regular and dense arrangement and a fewer number of condensed nuclei were observed after treatment with PNS (Figures 1(a)–1(e)). Moreover, the numbers of neurons in CA1 region in the model group remarkably decreased compared to the control group and PNS treatment significantly increased the number of neurons in CA1 region (Figure 1(f)). These results indicate that PNS plays a role in alleviating pathomorphological change and preventing neuronal loss.

It has been reported that 8-OHdG, which is the most commonly analyzed biomarker of oxidative damage to DNA, showed higher levels in the brains of AD patients than in subjects with normal aging [20]. In our study, we observed that the level of 8-OHdG in the model group increased compared to that of the control group, and PNS treatment retarded the production of 8-OHdG in AD mice (Figure 2(d)), suggesting that PNS holds the potential to alleviate DNA injury induced by oxidative stress.

Antioxidant enzymes such as SOD, CAT, and GSH-PX are critical in protecting neurons from oxidative damage. Our results demonstrated that the percentages of immunopositive neurons and activities of SOD, CAT, and GSH-PX were declined in the model group compared to the control group (Figures 2(a)–2(c)), which are consistent with the trend of the data published in this regard [21], and PNS treatment significantly increased the percentages of immunopositive neurons and activities of SOD, CAT, and GSH-PX in the brains of SAMP8 mice (Figures 2(a)–2(c)), suggesting that PNS enhances the expressions and activities of SOD, CAT, and GSH-PX, thereby improving antioxidant capacity.

It is generally believed that, under oxidative stress, cells activate cytoprotective mechanisms by enhancing the expression or function of UCPs, such as UCP4 and UCP5, which are the dominant isoforms of UCP expressed in brain, to increase survival [22], and thus UCP4 and UCP5 may have important roles in protecting neurons from oxidative stress damage in AD. However, to date, little had been reported concerning the expressions and activities of UCP4 and UCP5 in the pathogenesis of AD. In our present study, the results indicated that the levels of UCP4 and UCP5 mRNA were significantly increased in SAMP8 model mice relative to SAMR1 controls (Figures 2(e)–2(h)), suggesting that compensatory increases in UCP gene expression may be utilized as a cytoprotective mechanism to compensate for the increased level of oxidative stress in an early-stage of AD pathogenesis. In addition, our results demonstrated that PNS could enhance the expressions of UCP4 and UCP5 mRNA and protein (Figures 2(e)–2(h)) and thus may have the ability to protect neurons in AD brain from oxidative stress damage.

## 5. Conclusion

In the current study, we showed that PNS has the potential to protect neurons from oxidative damage via attenuating the production of 8-OHdG, enhancing the activities of antioxidant enzymes and the expression levels of UCP4 and UCP5 mRNA and protein in the SAMP8 mouse model of AD. Accordingly, PNS may be a promising agent for AD treatment.
